# Research: Prevalence of neural tube defects Khartoum, Sudan August 2014–July 2015

**DOI:** 10.1186/s13104-016-2298-6

**Published:** 2016-11-24

**Authors:** Ilham M. Omer, Osman M. Abdullah, Inaam N. Mohammed, Lina A. Abbasher

**Affiliations:** 1 Faculty of Medicine, University of Khartoum, Khartoum, Sudan; 2 Sudan Medical Specialization Board, Khartoum, Sudan; 3 Academy of Medical Science and Technology, Khartoum, Sudan

**Keywords:** Neural tubes defects, Newborn, Spinabifida, Meningeocele

## Abstract

**Background:**

Neural tube defects (NTDs) are birth defects that results from failure of the neural tube to develop properly during early pregnancy.

**Methods:**

We studied the prevalence of neural tube defects in newborns admitted to the NICU in Soba University and Omdurman Maternity hospitals, during the period 1st August 2014 to 31st July 2015. A cross-sectional hospital based study, involved all newborns with any type of neural tube defect admitted to the NICU in the study area during the study period. Data was collected using a questionnaire reviewing the medical, social history and clinical examination.

**Results:**

Out of the 36,785 delivered newborns during the study period, the prevalence of NTDs was 2.8:1000. Females were 56 (54.4%) predominated males 47 (45.6%). History of neural tube defects was found in 11 (10.7%) of the affected newborns siblings. Sixty-eight (66%) of the studied mothers received folic acid during pregnancy with the current child, of those who received folic acid 66 (97.1%) started folic acid after conception, 36 (54.5%) in the first trimester and 39 (57.4%) had no regular intake of the folic acid. The types of NTDs include myelomeningocele 49 (47.6%), anencephaly 18 (17.5%), encephalocele 14 (13.6%), myelomeningocele and hydrocephalus 11 (10.7%) and meningocele 8 (7.8%).

**Conclusion:**

The prevalence of neural tube defects is 2.8:1000. Myelomeningocele is the commonest encountered NTD. The use of preconception folic acid needs to be advocated.

## Background

An estimated 1 in 1000 infants are born with a neural tube defect (NTD) annually in the United States [1], and more are spontaneously aborted or electively terminated. The most common types of NTDs are anencephaly and spinabifida, which typically present as open NTDs; such NTDs occur when neural tissue is exposed to the environment or only covered by a membrane. Less common are encephalocele and meningocele, which typically present as closed NTDs, in which the defect is covered by normal skin. Anencephaly is fatal in all cases; infants with spinabifida frequently survive following surgery [1]. Although no single major gene has been implicated as causal in the development of NTDs [2], these defects are thought to result in part from genetic risk factors. Environmental (non-inherited) factors are also thought to play a role in NTD development; however established risk factors, such as folate levels [3, 4], maternal diabetes [5], and use of antiepileptic medications [6, 7], account for only a small proportion of prevalent NTDs, indicating that unidentified risk factors for NTD still remain.

The main cause of NTDs are abnormalities that occur during neurulation, which should be complete by 4 weeks postconception [8].

However, these serious birth defects are to a large extent preventable by adequate intake of folic acid by women of the reproductive age and several studies reported that preconceptional supplementation of folic acid can prevent up to 50% of the cases of NTDs as well as cardiac and craniofacial abnormalities [9–11]. Despite wide availability of its natural food sources (green leafy vegetables, bananas, legumes), folic acid deficiency among women of reproductive age is common worldwide [12, 13] usually as a result of low-dietary intake or cooking losses [14].

## Methods

### Study design

A cross -sectional, hospital based study.

### Study area

The two main neonatal care units in Khartoum state:

1. Soba university hospital NICU: Soba University Hospital (SUH) is located 15 km south of the center of the capital of Sudan, Khartoum, and considered the largest training hospital for the students of the faculty of medicine, University of Khartoum. The NICU in the hospital receives about 700-800 newborns yearly, all of them should be born in the hospital. Obstetric department in SUH is one of the main referral centers for high risk pregnancies in Sudan and receives cases from the whole country including both governmental and nongovernmental sectors.

The fetal unit in the hospital receives the referred critical pregnancies from all over the country.

(2) Omdurman maternity hospital: Has been established in 1957 as the first specialized hospital in the country, to provide care and medical services to mothers and newborns babies, its located in Khartoum state, Omdurman province. Hospital services cover all the surrounded area, rural areas and even nearby towns. The NICU department accommodates about 100 babies and admit babies born inside the hospital as well as those referred from other hospitals.

Home deliveries constitute about seventy percent of the total deliveries. Birth registry is established in hospitals and probably big cities and hence a lot of cases were missed.

Prenatal diagnosis is limited to few hospitals in the capital, Khartoum

Newborns with NTDs who were born in other hospitals or even at home were not captured in the study.

Both hospitals accept high risk pregnancies but the normal pregnancies constitute the majority.

### Inclusion criteria

All newborns with any type of neural tube defect presenting to the study area within the study period.

### Sampling Technique

Total coverage: including all newborns with any type of neural tube defect presenting to the study area within the study period.

### Data Collection Tools

Data was collected by questionnaire reviewing personal, medical, and social data and clinical examination findings of the newborn.

The questionnaire was completed by doctors only during the first 24 h after delivery.

### Statistical analysis

Data was analyzed using Statistical Packages for Social Sciences (SPSS) and the results was presented in forms of tables and graphs.

## Results

The total number of the infants delivered during the study period was 36,785, 103 of them were newborns with NTDs, hence the prevalence of NTDs = 103/36,785 = 2.8: 1000.

Sex distribution of the children in the study revealed that females 56 (54.4%) while males were 47 (45.6%) (Table 1).

**Table 1:** Characteristics of mothers and newborns

Characteristics	Cases	
	N	%
Gender		
Male	47	45.6
Female	56	54.4
Total	103	100.0
Parity		
PG	20	19.4
Multipra	57	55.3
Para II	13	12.6
POT I	1	1.0
Grand multipra	12	11.7
Total	103	100.0
Received folic acid		
Yes	68	66.0
No	35	34.0
Total	103	100.0
Timing		
Preconception	2	2.9
After conception	66	97.1
Total	68	100.0

Seventy-two (68.9%) of mothers were multipra and 12 (11.7%) were grandmultipra and 20 (19.4%) were primigravidae (Table 1).

The types of NTDs revealed in the study were myelomeningocele 49 (47.7%), anencephaly 18 (17.5%), encephalocele 14 (13.6%), myelomeningocele and hydrocephalus 11 (10.7%) and meningocele 8 (7.8%) (Table 2).

**Table 2:** The types of neural tube defects in relation to folic acid intake

Types	N	%	Received folic acid	No folic acid
Anencephaly	18	17.5	13	5
Encephalocele	14	13.5	11	3
Myelomeningocele	49	47.6	25	24
Meningocele	8	7.8	6	2
Spina bifida occulta	2	1.9	2	0
Myelomeningocele + Hydrocephalus	11	10.7	10	1
Meningocele + cleft lip + palate	1	1.0	1	0
Total	103	100.0	68	35

Chi square: 16.48; p value: 0.02

Regarding folic acid intake 68 (66%) of the studied mothers received folic acid during pregnancy with the current child, of those who received folic acid 66 (97.1%) started medication after conception, 36 (54.5%) started in the first trimester and 39 (57.4%) had irregular intake of folic acid. It should be noted that 35 (34%) of the mothers did not receive folic acid during pregnancy with the current child (Table 2).

The majority of the newborns 76 (73.8%) were referred to the paediatric surgery and 27 (26.2%) died of different causes.

## Discussion

One hundred and three newborns with neural tube defect were enrolled in the study to assess prevalence and short term outcome of NTDs in Soba University and Omdurman Maternity Hospitals.

The total number of the infants delivered in the study area during the study time was 36,785 babies. Hence, the prevalence of NTDs was 103/36,785 = 2.8: 1000. This near to the findings of the case–control study done by Elsheikh in Omdurman Maternity Hospital in which the incidence of NTDs was 3.48/1000 [13]. However, the prevalence in Jordan was estimated by Masri [14] to be 1.1/1000 among 28,301 live births,which similar to the prevalence reported by Busby et al. [15] in the United Kingdom and Ireland (1–1.5/1000 in the 1990s). High prevalence of NTDs in Sudan compared to other countries attributed to that termination is not considered after antenatal diagnosis [11] and the intake of folic acid by the mothers usually starts after conception due to lack of awareness of its importance [12, 13]. The country is under developed one and lack a lot of the resources and health programs and hence there is no routine checkup for the majority of the population including pregnant women and usually people seek medical advice late.

Prenatal diagnosis in Sudan can help in counseling the parents regarding the outcome and possible management but the issue of termination needs joint work between Islamic countries as Fatwa, the religious permission, is usually respected by people.

The prevalence of NTDs in this study does not reflects the actual community level as the majority of deliveries, more than 75%, are still conducted at home where most of the affected newborns die either of sepsis or lack of the needed medical care. The lack of referral system and easy transportation means from most of the remote areas play a major role in missing a lot of cases.

Sex distribution of the children in this study shows that females 56 (54.4%) were slightly predominated 47 (45.6%) males. This is similar to the findings of the study done by Larry and Paulozzi [16] and Forrester and Merz [11]), which showed that females are more likely than males to have anencephaly and spina bifida, with the difference greater for the latter defect. This higher rate among females appears to be influenced by the presence of additional birth defects, geographic, and other factors.

The common types of NTDs include myelomeningocele 49 (47.7%), anencephaly 18 (17.5%), encephalocele 14 (13.6%), myelomeningocele and hydrocephalus 11 (10.7%) and meningocele 8 (7.8%). This agreed with study conducted by Mohammad AL-Qudah et al. [17] about Neural Tube Defects at Prince Rashid BinAl-Hassan Hospital which revealed that during the study period, there were 17 cases of neural tube defects (1.4/1000 births), of these there were five cases of spina bifida (0.42/1000 births), three cases of encephalocele (0.25/1000 births), and nine cases of anencephaly (0.76/1000 births). The overall female to male ratio was 1:0.89. The most common neural tube defect was anencephaly (52.9%), and the commonest site of spina bifida was the lumbosacral region in 2/5 (40%). One case of encephalocele was associated with malformations while four cases (80%) of spina bifida were associated with other malformations [18].

The isolated NTDs constitute the majority of the studied patients which is different from the results obtained In a study from Riyadh [19] where syndromic, genetic (mainly inherited as autosomal recessive), and chromosomal defects were more prevalent than in other populations, and constituted around 20% of total NTDs.

 In a study done in 2010 by Lopez-Camelo et al. they found that folic acid fortification was associated with a decrease in the proportion of isolated cases and thereby an increase in the proportion of non-isolated cases. The higher proportion of non-isolated SB cases after fortification is consistent with previous reports, indicating that folic acid is most effective in reducing the occurrence of isolated spina bifida [13].

The short term outcome of the studied newborns revealed that 26.2% of them died of different causes including sepsis and other associated complications, while 73.8% were referred to the paediatric surgery for further management.

## Conclusion

- In this study the prevalence of NTDs was found to be 2.8: 1000 births.
- Most of the common types of NDTs were found among the studied newborns, with high rate of myelomeningocele followed by anencephaly.
- The short term outcome of NTDs included 27 deaths, which is about 26.2% of the newborns, the rest were referred to the paediatric surgery.

## Abbreviations

NICU: Neonatal intensive care units
NTD: Neural tube defects
PG: Primi gravida
SUH: Soba University Hospital
